# Spectroscopic Characterization of Mitochondrial G-Quadruplexes

**DOI:** 10.3390/ijms23020925

**Published:** 2022-01-15

**Authors:** Sara Illodo, Cibrán Pérez-González, Ramiro Barcia, Flor Rodríguez-Prieto, Wajih Al-Soufi, Mercedes Novo

**Affiliations:** 1 Departamento de Química Física, Facultade de Ciencias, Universidade de Santiago de Compostela, 27002 Lugo, Spain; sara.illodo.brea@usc.es (S.I.); danielcibran@gmail.com (C.P.-G.); wajih.al-soufi@usc.es (W.A.-S.); 2 Centro Singular de Investigación en Química Biolóxica e Materiais Moleculares (CiQUS), Departamento de Química Física, Universidade de Santiago de Compostela, 15782 Santiago de Compostela, Spain; flor.rodriguez.prieto@usc.es; 3 Departamento de Bioquímica e Bioloxía Molecular, Facultade de Veterinaria, Universidade de Santiago de Compostela, 27002 Lugo, Spain; ramiro.barcia@usc.es

**Keywords:** DNA quadruplexes, G-quadruplex CD and fluorescence spectroscopy, G-quadruplex structure

## Abstract

Guanine quadruplexes (G4s) are highly polymorphic four-stranded structures formed within guanine-rich DNA and RNA sequences that play a crucial role in biological processes. The recent discovery of the first G4 structures within mitochondrial DNA has led to a small revolution in the field. In particular, the G-rich conserved sequence block II (CSB II) can form different types of G4s that are thought to play a crucial role in replication. In this study, we decipher the most relevant G4 structures that can be formed within CSB II: RNA G4 at the RNA transcript, DNA G4 within the non-transcribed strand and DNA:RNA hybrid between the RNA transcript and the non-transcribed strand. We show that the more abundant, but unexplored, G6AG7 (37%) and G6AG8 (35%) sequences in CSB II yield more stable G4s than the less profuse G5AG7 sequence. Moreover, the existence of a guanine located 1 bp upstream promotes G4 formation. In all cases, parallel G4s are formed, but their topology changes from a less ordered to a highly ordered G4 when adding small amounts of potassium or sodium cations. Circular dichroism was used due to discriminate different conformations and topologies of nucleic acids and was complemented with gel electrophoresis and fluorescence spectroscopy studies.

## 1. Introduction

The discovery of the DNA double helix structure in 1953 led to an enormous growth in our understanding of nucleic acids and their biological functions. However, DNA does not only display single- or double-stranded structures [1,2]. For example, conformations, such as Holliday junctions, replication forks and DNA flaps, are formed during DNA replication, recombination and repair processes. Moreover, specific DNA sequences can also display a number of different conformations, such as i-motif structures, hairpins and especially guanine quadruplexes, which have gained the attention of many researchers due to the recent evidence of their important biological implications. Guanine quadruplexes (G4s) are currently known to be involved in key processes, such as transcription, replication and telomeric inhibition, and a variety of disorders, such as genome instability and cancer [3,4,5].

**Figure 1:**
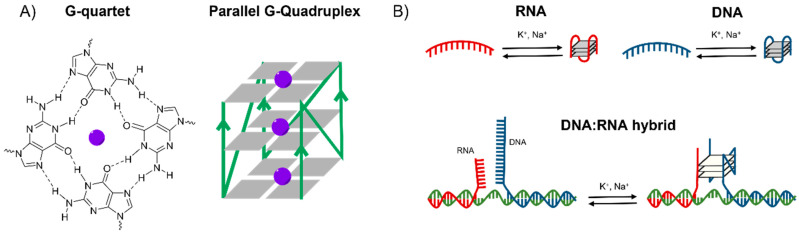
(**A**) Chemical structure of a G-quartet and schematic representation of a parallel G-quadruplex (G4). (**B**) Schematic representation of the three distinct G4s that can be formed within mitochondrial CSB II: unimolecular RNA G4 assembly adopted within the RNA transcript, unimolecular DNA G4 formed within the non-template strand and bimolecular DNA:RNA G4 hybrid formed between the non-template DNA and the RNA transcript.

G4s appear on single stranded DNA and RNA guanine-rich regions, forming a highly polymorphic four-stranded structure. Its primary unit, the G-quartet, is a square planar assembly of four guanines that forms Hoogsteen hydrogen bonds (Figure 1A) [3,4,5,6]. The union of two or more G-quartets conforms the quadruplexes, which can be both intramolecular and intermolecular. It is known that G-quartet’s stacks are stabilised by cations coordinating with the guanine O6 atoms (purple spheres in Figure 1A), preferably with monovalent cations (stability K^+^ > Na^+^ > Li^+^) that are also the most relevant at physiological conditions [7,8,9,10,11]. G4 structures can adopt different topologies, which are classified on the basis of the combination of strand direction as parallel (Figure 1A), antiparallel and hybrid, and are dependent of the strand length and loop composition [12,13,14,15,16,17,18].

The formation of G4 on a given nucleic acid sequence can be predicted if the motif G_≥3_N_1-7_G_≥3_N_1-7_G_≥3_N_1-7_G_≥3_ is present, where N represents the loop sequence and can be any nucleobase, including guanine. The first computational studies that used this motif estimated over 350,000 sequences prone to G4′s formation within the human genome [3]. Later computational and sequencing studies reported more than 700,000 potential G4-forming sequences present in the human genome [19,20]. G4 formation in vivo [21] has also been reported as well as its presence in cytoplasm [22,23,24], mitochondria [25,26], nucleoli [27,28] and nuclei [29], and on functional regions, such as telomers [30,31], gene promoters, 5′ untranslated regions and splicing sites [19,20].

In recent years, reports on G4 formation in mitochondria have led to an increased interest in these structures. The replication mechanism of human mitochondria requires a ~120-nt RNA transcript that is formed at the G-rich conserved sequence block II [32]. It has been suggested that three possible G4s can appear at mitochondrial CSB II (Figure 1B): (1) an unimolecular RNA G4 assembly adopted co-transcriptionally within the RNA transcript; (2) an unimolecular DNA G4 formed within the non-template strand; and (3) a bimolecular DNA:RNA G4 hybrid formed between the non-template DNA and the RNA transcript with a potential role in R-loop stabilisation [12,13,15].

The important biological implications of CSB II have attracted the interest of researchers, who have focused on the study of the transcription termination mechanism [26,33,34,35,36,37,38]. Most studies focused on the G5AG7 sequence, which only constitutes a 13% of CSB II sequences, G6AG7 (37%) and G6AG8 (35%) being the most common [36]. Biophysical studies of the G5AG7 DNA, RNA and hybrid G4s have shown a characteristic parallel shape in CD experiments, although polyacrylamide gel electrophoresis has revealed the presence of *two different* G4 conformations for the RNA sequence [12]. Additionally, Hillen and collaborators, who studied the G6AG8 sequence, reported that G4 formation involves a guanine located 1 bp upstream the G6AG8 sequence [13].

Despite the clear evidence of G4 formation in mitochondria, no conformational analysis of G6AG8 (and G6AG7) has been reported to date. We must take into account that a higher number of guanines would increase G4 stability, and therefore increase transcription termination [35,36,37]. To better understand the role of the most common G4s found in mitochondria in transcription termination and R-loop stabilization, it is necessary to elucidate their folding mechanism, conformational dynamics and relative stability.

In this study, we aim to characterize all three G4 structures from the CSB II mitochondrial region using circular dichroism and time-resolved fluorescence techniques. The three sequences at CSBII that may be capable of forming G4 (G5AG7, G6AG7 and G6AG8) were studied, as well as their analogous sequences GCG6AG5, GCG6AG7 and GCG6AG8, to clarify the role of the guanine located 1 bp upstream on the G4 formation (see Supplementary Table S1). Circular dichroism is the main technique used in this study, as it can decipher if a given sequence is folding into G4 or a different secondary structure and, in addition, discriminate between the different topologies adopted when folding into G4 [17,39,40]. Furthermore, gel electrophoresis and fluorescence measurements were performed to reinforce the results.

## 2. Results and Discussion

### 2.1. Circular Dichroism

#### 2.1.1. DNA Quadruplexes: Influence of the Sequence

**Figure 2:**
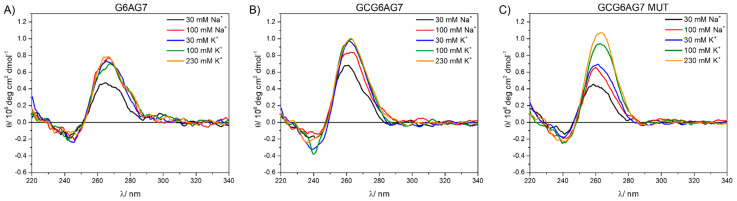
Circular dichroism spectra for DNA sequences G6AG7 (**A**), GCG6AG7 (**B**), and GCG6AG7 MUT (**C**) in a phosphate-buffered solution with cations Na^+^ and K^+^ in the concentration range from 30 mM to 230 mM.

We used CD measurements to investigate the formation of quadruplexes by the sequences under study. For this purpose, CD spectra were acquired for the DNA sequences with the guanine 1 bp upstream (GCGXAGY) and without it (GXAGY) and all three lengths of the chains (see Supplementary Table S1 for details). These spectra are shown in Figure 2 for the X–Y = 6–7 and in Supplementary Figure S1 for all three lengths (X–Y = 5–7, 6–7 and 6–8). For all sequences, two bands are observed, one negative band with minimum at about 245 nm and another intense, positive band at around 265 nm. The positions of these two bands coincide with those reported for parallel G4 conformations [17]. Moreover, the CD spectra do not show significant bands at wavelengths higher than ~290 nm, which would be characteristic of both the antiparallel and the hybrid topologies. This result demonstrates that parallel G4s are formed by all sequences GCGXAGY and GXAGY.

Even though all studied sequences show CD bands that are coincident with the reported CD bands for parallel G4 conformations, the comparison between GXAGY and GCGXAGY sequences shows subtle differences. GXAGY spectra present lower intensities in all studied media than their analogous GCGXAGY sequences (compare Figure 2A with Figure 2B and the upper row with the lower row in Supplementary Figure S1). In addition, GCGXAGY sequences have better defined CD bands, and their maxima show a slight shift to the red with respect to the former (Figure 2B and Supplementary Figure S1D–F). These significant differences observed between GXAGY and GCGXAGY CD spectra suggest that the additional upstream guanine actually plays a key role in the G4 formation. Its important role was further corroborated by studying the sequence GCG6AG7 MUT, where some guanines were replaced by adenines (see Supplementary Table S1). As shown in Figure 2C, this mutant sequence shows CD spectra very similar to those of GCG6AG7, with identical positions of the bands and similar intensities, indicating that the guanine present 1 bp upstream is more important for G4 formation than the lower number of guanines in the chain. These observations suggest that the additional guanine is part of the G4 structure. However, the stronger dependency with cation concentration of GCG6AG7 MUT indicates a lower stability of G4s in this mutant sequence in comparison to GCG6AG7. Applying the same argument to the other sequences, we can conclude that all studied sequences of the CSB II form parallel G4s, which are favored when a guanine is present 1 bp upstream.

**Figure 3:**
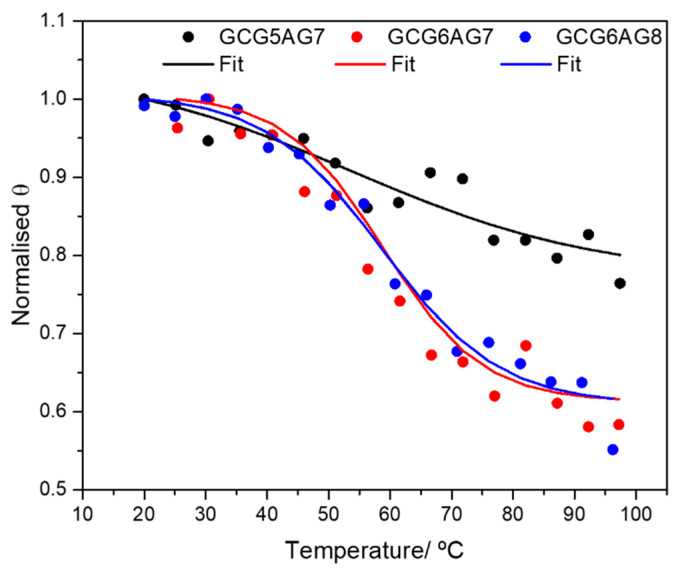
CD melting data: normalized value of the ellipticity Θ at the maximum of the CD spectra as a function of temperature and Boltzmann fits to the experimental data for DNA sequences GCG5AG7 (black dots), GCG6AG7 (red triangles) and GCG6AG8 (blue squares) in a phosphate-buffered solution with 30 mM of K^+^. The corresponding fitted melting temperatures are given in Table 1.

Additionally, Supplementary Figure S1 shows the influence of the number of guanines: as compared to the longer sequences, the CD bands of the shorter ones (G5AG7 in Figure S1A, and GCG5AG7, Figure S1D) have less intensity and show a negligible spectral shift as the cation concentration is increased, suggesting a less effective G4 formation, even when the guanine 1 bp upstream is present. The results of CD melting experiments confirm this interpretation. Figure 3 shows the melting data for the three different GCGXAGY sequences in a phosphate-buffered solution with 30 mM of K^+^, fitted using Boltzmann’s equation to obtain the melting temperature (*T_m_*). The corresponding CD spectra are shown in Supplementary Figure S2A–C. The small variation with temperature together with a poorly defined *T_m_* of the shorter sequence (Supplementary Figure S2A, black dots in Figure 3 and Table 1) confirm its exiguous G4 stability. Instead, the longer sequences show typical melting curves, with identical melting temperatures around 59 °C. This value, in very good agreement with those reported in the literature for different G4s of parallel topology [41], confirms the stability of the G4s for the longer DNA sequences. Moreover, melting experiments at higher cation concentrations of these longer sequences GCG6AG7 and GCG6AG8 (see Supplementary Figure S2D for GCG6AG7 at 100 mM K^+^) show practically no variation of the CD spectrum, proving the increased stability of the quadruplexes formed by these sequences at near-physiological cation concentrations.

Thus, all these findings lead to the conclusion that the longer sequences, GCG6AG7 and GCG6AG8, which are also more abundant in the CSB II, have a higher tendency to form quadruplexes. In consequence, from this section onwards, we focus our study on those longer sequences.

#### 2.1.2. DNA Quadruplexes: Influence of the Cation

CD spectra in Figure 2 and Supplementary Figure S1 show that the nature of the cation (Na^+^ or K^+^) and its concentration have a significant effect both in the intensity and in the position of the bands. Thus, G4 formation seems to be favored by higher cation concentrations, and more by the cation K^+^ than by Na^+^.

To gain a deeper insight into the influence of the cation concentration on G4 formation, we changed the buffer to one that does not contain sodium or potassium cations itself, so that samples without these cations and lower cation concentrations could be obtained. Hence, we measured additional CD titrations for the GCG6AG7 sequence in a Tris buffer varying the concentrations of sodium or potassium (Supplementary Figure S3A,B). In the absence of sodium or potassium, the CD spectrum of GCG6AG7 shows the two previously observed bands. The intensities of these bands increase significantly with the addition of very low concentrations of the cations, especially with K^+^. This increase is associated with a slight, but noticeable shift of the positive band towards longer wavelengths, suggesting a change of topology correlated with the presence of the cations.

**Figure 4:**
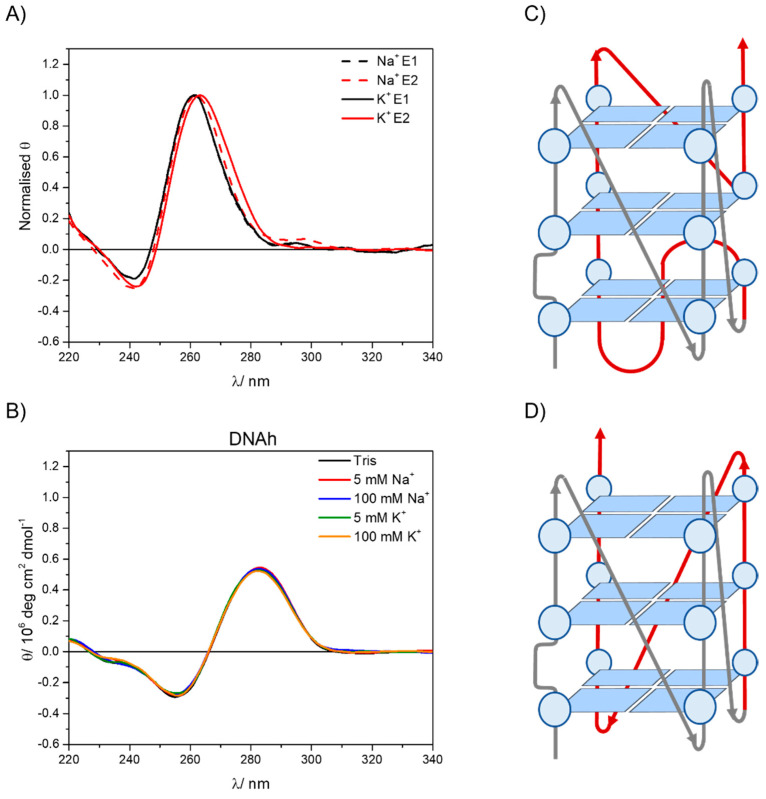
(**A**) Normalized pure spectra obtained from the non-linear global analysis of the CD titration data for the GCG6AG7 sequence with potassium (black lines) and sodium (dashed lines) using Hill’s equation. (**B**) CD spectra of DNAh in a Tris buffer and different added sodium and potassium concentrations. (**C**) Proposed structure for the less ordered, (−p−p−p+p) parallel G4. (**D**) Proposed structure for the highly ordered, −(ppp) parallel G4.

In order to extract as much information as possible from the CD titrations, PCA combined with GA analysis were applied. PCA reveals that the experimental CD spectra are contributed by two different species. Using Hill’s model (Equation (S3) in the Supplementary Data) to fit the variation of the experimental data (Supplementary Figure S3C) by GA, the “pure” spectra related to those two species were obtained. These spectra are shown in Supplementary Figure S3D with their real intensities and in Figure 4A normalized for a better comparison, E1 (black lines) being the CD spectrum of the GCG6AG7 sequence in the absence of cation and E2 (red lines) being the CD spectra of GCG6AG7 with sodium (dashed red line) and potassium (solid red line).

The pure spectra resulting from this analysis show that, even in the absence of sodium or potassium cations, the GCG6AG7 sequence forms parallel G4s. Nevertheless, a slight shift of the two CD bands to the red is associated with the addition of the cations, more significantly in the case of potassium, which coincides with that reported for parallel G4s of two topologies with different types of loops [17]. Following the folding topologies described by Karsisiotis and collaborators, we propose that, in the absence of cations, GCG6AG7 forms a less ordered, parallel G4 with looping sequence (−p−p−p+p) (Figure 4C), which reorganizes in the presence of the cations, yielding a highly ordered, more stable G4 of topology −(ppp) (Figure 4D). The given looping sequences follow the convention of a positive sign (+) for tetrad loops progressing clockwise and negative sign (−) for those progressing counter-clockwise. The letter *p* stands for the type of the loop being a propeller [17]. For sodium cation, a less significant shift is observed (Figure 4A), which might be due to an incomplete rearrangement of the G4. The small apparent peak observed around 295 nm is attributed to an artefact resulting from the treatment of the noisy raw CD spectra, including baseline correction and smoothing.

The titrations showed that only small concentrations of the cations are needed to bring about the reported G4 rearrangement, especially in the case of potassium. The fits of the Hill equation to the titration data also yield an estimation of the relative stability of the two G4s, based on the value of the rearrangement constant (*K*), which corresponds to the equilibrium shown in Equation (S2) of Supplementary Materials backwards, that is, the change from the ordered G4_f_ to the less ordered G4_i_. The values obtained for *K* are 0.34 ± 0.06 mM with potassium and 2.8 ± 0.4 mM with sodium, with a Hill’s coefficient of about 1, which shows no cooperativity of the binding process. Hence, the ordered G4_f_ is better stabilized by potassium than by sodium and needs only a very small cation concentration to form. This behavior is in accordance with that reported for other G4s [9,11].

To rule out the potential contribution of other DNA conformations in the sequences under study, CD spectra were measured for a DNA without any guanine-rich sequence (DNAh, Supplementary Table S1) that presumably cannot form G4. Comparing these spectra (Figure 4B) with those of guanine-rich sequences (Figure 2 and Supplementary Figure S1), the differences between non-G4 and G4 CD bands are clear, the positions of the bands being significantly red shifted for the non-G4 DNA (negative band at about 255 nm and positive band at about 285 nm). Moreover, no change of the CD spectra is observed with cation type and concentration, confirming that the features observed in the CD spectra of guanine-rich sequences are due to the formation of G4 conformations.

#### 2.1.3. DNA, RNA and DNA:RNA Hybrids Quadruplexes

**Figure 5:**
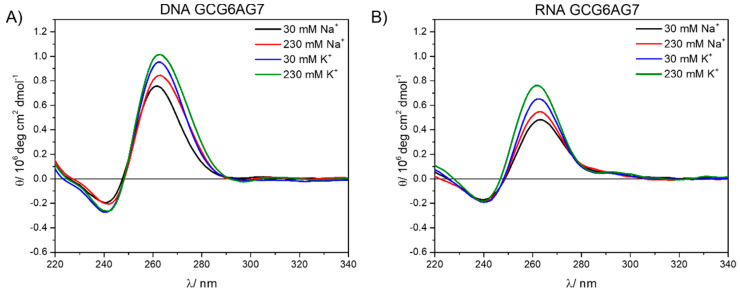
DNA (**A**) and RNA (**B**) circular dichroism spectra at 30 mM and 230 mM of sodium (black and red) and potassium (blue and green).

When comparing same length RNA (rGCGXAGY) and DNA (GCGXAGY) sequences under the same cation concentration conditions, we observe similar patterns in the CD spectra (Figure 5 and Supplementary Figure S4). This confirms that these RNA sequences also form quadruplexes and with a parallel topology. The positions of the CD bands correspond to the highly ordered G4, which is formed in a phosphate buffer even without the addition of salt. However, RNA sequences show lower intensities for all sequences and cation concentrations than DNA, suggesting a lower stability of the RNA-G4s (see Supplementary Figure S4 for comparison of all sequences). Additionally, a very low but persistent positive band is observed around 290 nm for all three rGCGXAGY sequences, which is not present in the corresponding DNA sequences and might be due to the formation in a small extent of antiparallel and hybrid topologies [17].

**Figure 6:**
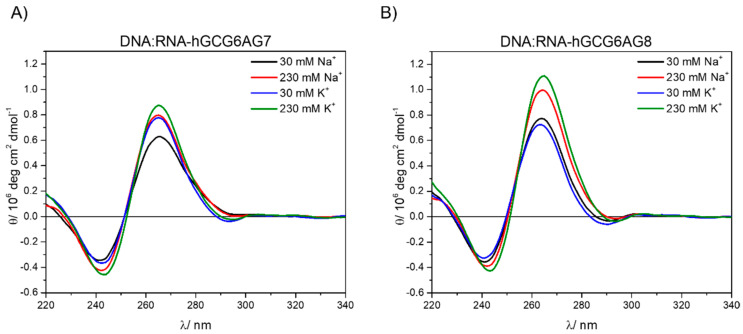
CD spectra of the hybrids DNA:RNA-hGCG6AG7 (**A**) and DNA:RNA-hGCG6AG8 (**B**) in a phosphate buffer at different concentrations of sodium and potassium after removing the contribution of non-G4 DNA (see Supplementary Figure S5).

Finally, the CD spectra of the DNA:RNA hybrids with the longer sequences, DNA:RNA-hGCG6AG7 and DNA:RNA-hGCG6AG8 (see Table S1 and following text) were measured. Raw data showed very broad bands (Figure S5) that were obviously contributed by two species: the G4 and the non-G4 DNA that appears due to an incomplete hybridization between DNA and RNA chains. Since the CD spectrum of the non-G4 DNA (DNAh) was known (Figure 4B), it was possible to remove this contribution following the method explained in the Supplementary Materials (Figure S5) and to restore the CD spectra of the hybrid alone (Figure 6). These CD spectra show similar patterns as the DNA and RNA sequences, confirming the formation of parallel quadruplexes in these hybrid forms with similar stability as for DNA sequences. No further bands are observed at higher wavelengths suggesting the formation of pure parallel G4s. DNA–RNA hybrid G4s formed by telomeric DNA and RNA have been recently reported with parallel conformation and similar melting temperature both in vitro and in the environmental conditions of HeLa cells [42].

**Figure 7:**
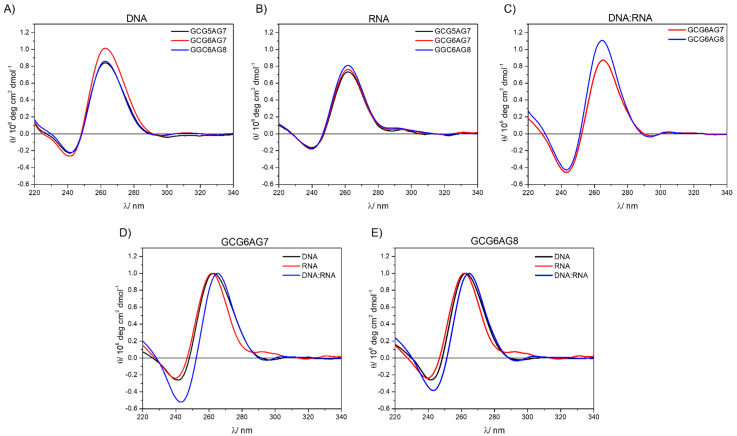
(**A**) CD spectra for different length DNA sequences at 230 mM K^+^; (**B**) CD spectra for different length RNA sequences at 230 mM K^+^; (**C**) CD spectra for different length DNA:RNA hybrids at 230 mM K^+^; (**D**) normalized CD spectra of GCG6AG7, rGCG6AG7 and DNA:RNA-hGCG6AG7; (**E**) normalized CD spectra of GCG6AG8, rGCG6AG8 and DNA:RNA-hGCG6AG8.

Figure 7 summarizes the results obtained from CD measurements, allowing the comparison among the three forms of quadruplexes under study (DNA, RNA and DNA:RNA hybrid) and the three lengths of sequences. The CD spectra show very similar bands in all types and sequences, typical for parallel G4. In the case of DNA:RNA hybrids, the observed slight shift to the red might be due to a somewhat different topology, but it can also be explained by a residual contribution of the non-G4 DNA. The comparison among the three different types of G4s (Figure 7D,E) show that, only in the case of RNA, there might be present small proportions of antiparallel or hybrid topologies, characterized by a positive band at about 290 nm.

On the basis of these results, we can conclude that all three possible forms of quadruplexes in the CSB II, DNA, RNA and DNA:RNA hybrid fold into parallel G4s with any sequence length, but more efficiently in GCG6AG7 and GCG6AG8, which are more abundant in the CSB II. The stability of the G4s increases with the addition of small cation concentrations, especially potassium.

### 2.2. Gel Electrophoresis

**Figure 8:**
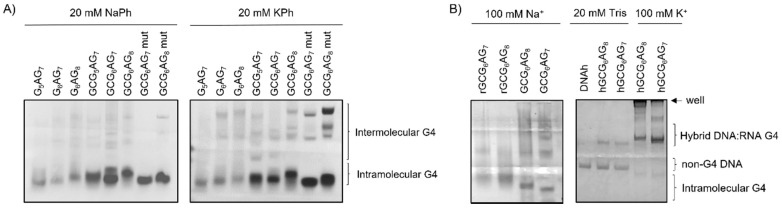
(**A**) Polyacrylamide gel electrophoresis developed with ThT for different DNA sequences in 20 mM sodium phosphate and 20 mM potassium phosphate (cation concentrations = 30 mM); (**B**) polyacrylamide gel electrophoresis developed with ThT for RNA (rGCGXAGY) and DNA (GCGXAGY) sequences in 100 mM Na^+^ and for DNAh and DNA:RNA (DNA:RNA-hGCGXAGY) sequences in 20 mM Tris (no cation) and 100 mM K^+^.

Polyacrylamide gel electrophoresis (PAGE) experiments were carried out in order to confirm G4 formation. Gels with all DNA sequences (GXAGY, GCGXAGY and GCGXAGY MUT) were prepared using sodium and potassium phosphates (cation concentration of 30 mM) and were developed with ThT, a fluorescence probe that is presumed to bind selectively to G4 DNA [43,44,45,46] (Figure 8A). These gels show fluorescent bands for all sequences that can be attributed to the G4s. The observed migration heights are in accordance with their molecular weights, which differ in 1 or 2 bases. However, the GXAGY sequences, without a guanine 1 bp upstream, present much lower intensities, in line with their lower efficiency of quadruplex formation. In the case of GCGXAGY mutants, additional upper bands are observed with very short migration, which can be attributed to the formation of intermolecular G4 structures. Thus, the fact that all studied sequences can fold into G4 structures is reinforced as well as the important influence of the first guanine in the G4 formation.

We also carried out gel electrophoresis for RNA sequences (rGCGXAGY) and DNA:RNA hybrids (DNA:RNA-hGCGXAGY), as well as for the non-G4 sequence DNAh. Figure 8B shows these gels together with those of the corresponding DNA sequences for comparison. It is observed that RNA sequences do not show clear bands, what is attributed to the lack of RNAase free conditions. Nevertheless, the wide bands of higher intensity present similar migration as the corresponding DNA sequences (Figure 8B, left).

DNA:RNA hybrid sequences show two different bands whose intensity change with the addition of potassium cations, the upper bands being much more intense in presence of potassium. Comparing the positions of the DNA:RNA-hGCGXAGY bands and their retention factor (Rf, Table S2) with those of the corresponding DNA sequences, we can conclude that the upper bands that increase intensity when adding salt correspond to the G4s formed after the hybridization of the three strands, which show much lower Rf than the corresponding DNA sequences due to their higher mass. The lower band, which is much more intense in the absence of salt, presents identical migration as the non-G4 DNAh and is therefore attributed to the residual DNAh strands from the incomplete hybridization.

In Figure 8B, we can see that all sequences present bands even though we are staining with ThT, which has been reported as an optimum probe for G4 recognition [43,44,45,46]. Gels stained first with Sybr Gold and then, after washing out, with ThT (Supporting Figure S6) show the same bands, but ThT clearly shows much less intensity with non-G4 than with G4 structures. As shown in the next section, fluorescence measurements prove that ThT binds also to some extent to non-G4 sequences, although the emission intensity is significantly lower than with G4 sequences. These results are also supported by other previous fluorescence studies of the fluorophore [47,48,49,50,51].

### 2.3. Fluorescence Measurements

In order to obtain further insight into the influence of the cation concentration in the G4 formation as well as to prove the binding of ThT to non-G4 structures, fluorescence measurements were performed. The advantage of ThT is its negligible emission in an aqueous solution in contrast to its high brightness when bound to proteins or other macromolecules. However, its complex photophysical behavior may complicate the interpretation of the observed fluorescence properties [52].

Steady-state and time-resolved fluorescence emission and anisotropy of ThT in the presence of DNA GCG6AG7 were measured as a function of potassium concentration and compared with those of ThT in presence of DNAh with and without 100 mM of potassium. For all these experiments, perchlorate salts were used instead of chlorides to prevent any possible quenching of the fluorescence. As pointed out above, we can assume that the observed fluorescence is only due to ThT bound to the DNA. We will denominate ThT bound to GCG6AG7 also as ThT:GCG6AG7 complex and ThT bound to DNAh as ThT:DNAh complex.

**Figure 9:**
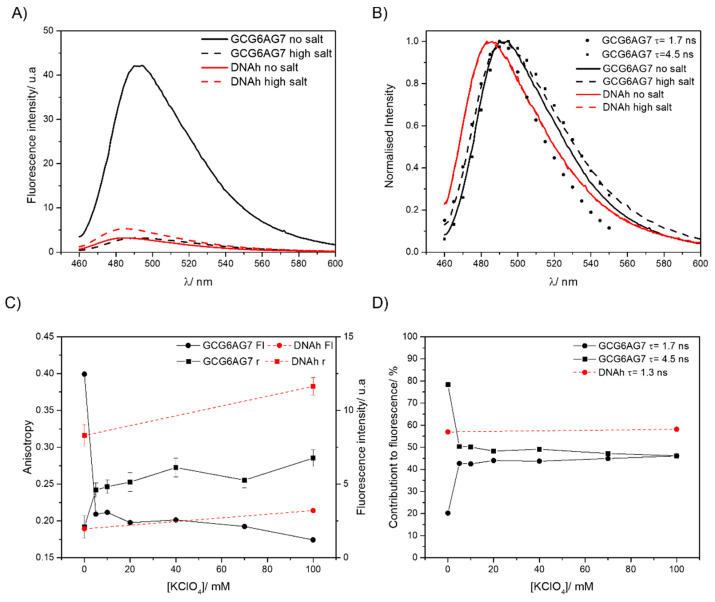
(**A**) Fluorescence emission spectra of ThT with GCG6AG7 and DNAh sequences in a Tris buffer in the absence of cations (solid lines) and with 100 mM potassium (dashed lines). (**B**) Normalized fluorescence emission spectra of graph A compared with the time-resolved emission spectra of the ThT species with τ = 1.7 ns and τ = 4.5 ns. (**C**) Steady-state fluorescence anisotropy (squares, left scale) and fluorescence intensity (circles, right scale) of ThT with GCG6AG7 (black symbols) and with DNAh (red symbols) sequences in a Tris buffer with different concentrations of potassium. (**D**) Percentage contributions to fluorescence of the two ThT species observed with GCG6AG7 (black symbols) and the one with DNAh (red symbols) in a Tris buffer with different concentrations of potassium.

Fluorescence emission spectra are shown as absolute intensities in Figure 9A and normalized in Figure 9B. In the absence of the cation, ThT bound to GCG6AG7 has about tenfold higher intensity than the ThT bound to the non-G4 DNAh. The addition of potassium causes a sharp decrease in the fluorescence intensity of the ThT bound to GCG6AG7, whereas in the case of the ThT bound to DNAh, it increases slightly (Figure 9A,C). The normalized spectra of the emission spectra of the ThT bound to the non-G4 DNAh (Figure 9B) show a considerable blue shift as compared to those of the ThT bound to the G4. These findings confirm that ThT is not specific for G4s, but unveil the distinct fluorescence properties of ThT when bound to GCG6AG7 or to DNAh.

Time-resolved fluorescence measurements allow us to identify the number and type of ThT species responsible for the observed emission. When bound to GCG6AG7, ThT shows two main lifetimes, which correspond to two different species of the ThT:GCG6AG7 complex (Table 2). The one with the longer lifetime of about 4.5 ns is the main species in the absence of cation, explaining 80% of the total fluorescence (black squares in Figure 9D), and its contribution decreases sharply to about 50% with the addition of potassium. On the contrary, the ThT:GCG6AG7 complex with the shorter lifetime of 1.7 ns contributes only 20% without potassium and around 45% in the presence of the cation (black circles in Figure 9D).

**Figure 10:**
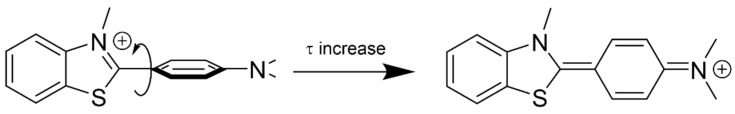
Schematic representation of the two limit conformations adopted by ThT [52].

ThT presents a wide distribution of ground-state conformations with different fluorescence properties, going from those with nearly perpendicular benzothiazole and aminobenzene moieties (Figure 10, left), characterized by blue-shifted fluorescence, low quantum yield and a short lifetime, to configurations that are almost planar (Figure 10, right), with red-shifted fluorescence, high quantum yield and long lifetime [52]. On this basis, the ThT bound to GCG6AG7 with the longer lifetime, predominant in the absence of cation, corresponds to a virtually planar conformation, explaining the high fluorescence intensity under such conditions (Figure 9A,C). Instead, the ThT bound to GCG6AG7 with the shorter lifetime presents some degree of torsion and is responsible for the lower, blue-shifted fluorescence observed in the presence of potassium (Figure 9A–C). The time-resolved fluorescence spectra obtained for these two types of bound ThT (black symbols in Figure 9C) are in agreement with this explanation.

Further insight into the conformations of ThT bound to GCG6AG7 is provided by fluorescence anisotropy measurements, which give information about the mobility of the fluorescent probe within its microenvironment. The steady-state fluorescence anisotropy value (Figure 9C) of the ThT bound to GCG6AG7 increases significantly as the potassium concentration is increased, indicating a decrease in the mobility of ThT when the cation is added. This can be explained on the basis of the rotational correlation times obtained in the time-resolved anisotropy measurements (Table 2). In the absence of potassium, most ThT molecules live enough in the excited state (4.5 ns) to be able to rotate to some extent with the corresponding rotational time of 3.20 ns. Instead, in the presence of potassium the rotational correlation time is higher (5.6 ns) and ThT molecules do not stay enough time in the excited state to rotate and depolarize.

Nevertheless, the most important result of these fluorescence data is the confirmation of the rearrangement of the DNA GCG6AG7 quadruplex in the presence of low amounts of cation, as observed in CD measurements. The sharp variations of the fluorescence intensity, steady-state anisotropy and lifetimes’ contributions (Figure 9C,D) with the addition of potassium support this hypothesis, showing that ThT must adapt to the topology of the G4.

Finally, fluorescence measurements show that ThT bound to DNAh presents very different fluorescence properties: much lower fluorescence intensity that increases slightly when potassium is added (Figure 9A,C), significant blue-shift of the emission spectrum (Figure 9B), shorter lifetime (Table 2) and high anisotropy due to the ineffective rotation during the lifetime (Figure 9C and Table 2). These results prove a higher degree of torsion between the moieties of ThT when bound to a non-G4 DNA (Figure 10) due to the different microenvironment of the probe.

## 3. Materials and Methods

### 3.1. Materials

DNA and RNA sequences were purchased from Integrated DNA Technologies (Coralville, IA, USA) and Biomers (Ulm, Germany), and dissolved in water, purified using a Millipore Milli-Q system, to obtain stock solutions at concentrations of the order of 100 µM. All studied sequences and their given abbreviations are shown in Supplementary Table S1. Thioflavin T (ThT, Merck, Darmstadt, Germany) was used without further purification. Sodium and potassium monohydrogenphosphate and dihydrogenphosphate salts were used to prepare the phosphate buffer (20 mM, pH 7.0) and Trizma hydrochloride for the Tris buffer (20 mM, pH 7.5). Sodium and potassium chlorides were added to adjust the desired cation concentration in the sample solutions. For the fluorescence measurements, sodium and potassium perchlorates were used instead of chlorides to avoid quenching. All these reagents were ACS grade (Merck, Darmstadt, Germany).

### 3.2. Sample Preparation

All samples for circular dichroism and fluorescence measurements were prepared under the same conditions. First, sample solutions were prepared in the phosphate or Tris buffer with a concentration about 6 µM of the nucleic acid and 0-200 mM of the desired cation. The samples were then heated in an Eppendorf ThermoMixer (Hamburg, Germany) at 95 °C for 15 min and then cooled with ice for another 15 min to achieve a fast-cooling process and avoid the formation of intermolecular structures. It must be noted that, when using the phosphate buffer, the cation concentration was already 30 mM without the further addition of salt.

### 3.3. Circular Dichroism

Regular circular dichroism (CD) spectra were acquired on a Jasco-715 spectropolarimeter (Hachioji, Japan) at 20 °C in a 2 mm cuvette. Melting experiments were performed under phosphate buffer conditions with a 2 mm cuvette and a temperature range from 20 to 95 °C on a Jasco-1100 spectropolarimeter (Hachioji, Japan). The absorption spectra of the samples were also recorded to determine their concentrations for the correction of the CD spectra.

### 3.4. Gel Electrophoresis

Electrophoresis experiments were carried out with 14% polyacrylamide gels on 0.5 X TB buffer (0.05 M Tris borate). Samples were prepared as exposed above for a final volume of 30 µL and 0.3 nmol of nucleic acid. Before loading the samples, 10 µL of 40% saccharose were added to them.

Gels were run for 1–2 h at 80–90 V with 0.5 X TB buffer and developed using ThT, as it was previously reported to be a selectively fluorescence probe that binds to G4 DNA [43,44,45,46]. Gel evolution was followed using bromophenol blue.

### 3.5. Fluorescence Measurements

Samples were prepared following the explained protocol and then ThT was added into the cuvette to achieve a 12 µM ThT concentration. Fluorescence emission spectra and steady-state fluorescence anisotropy were recorded using a F900 fluorimeter from Edinburgh Instruments (Livingstone, UK).

Fluorescence decays for lifetime and time-resolved anisotropy measurements were recorded using the time-correlated single-photon counting technique in an Edinburgh Instruments LifeSpec-ps time-resolved spectrometer (Livingstone, UK) with a 445 nm picosecond diode laser for excitation.

### 3.6. Data Analysis

All data were processed using the program OriginPro 19 (Origin Lab Corporation, Northampton, MA, USA). The presented circular dichroism spectra result from several corrections. First, baseline correction and blank subtraction were applied to the experimental ellipticity $\theta_{\exp}$. The resulting corrected spectrum was then divided by the nucleic acid concentration to obtain the molar ellipticity in deg cm^2^ dmol^−1^: $\theta \;=100\;\theta_{\exp}/C\;l$, being $\theta_{\exp}$ in deg, *l* in cm and *C* in mol dm^−3^. Only moderate smoothing was applied to these corrected spectra in order to prevent any possible distortion. Thus, CD spectra show more noise than usual.

Data from circular dichroism titrations, melting experiments and time-resolved fluorescence titrations were analyzed using a program developed by our group that carries out principal components analysis (PCA) to decipher the number of chemical species contributing to the experimental data and non-linear global analysis (GA) to fit the suitable model function to the experimental data [53]. The model functions used for each type of data are described in the Supplementary Materials. Time-resolved anisotropy decays were calculated using Equation (S1) shown in the Supplementary Data.

## 4. Conclusions

The detailed analysis shown in this work evidences the formation of G4 structures with parallel topology for the three G-rich main sequences within mitochondrial CSB II, those with a higher number of guanines and a guanine located 1 bp upstream being more stable. We observed a rearrangement of the G4 topology to a highly ordered structure with the addition of small amounts of cation, especially potassium. All three potential G4s form within CSB II: RNA G4 at the RNA transcript, DNA G4 within the non-transcribed strand and DNA:RNA hybrid between the RNA transcript and the non-transcribed strand. These findings contribute to a better knowledge of the G4 conformations found in mitochondria, which are thought to have a relevant role in transcription termination and R-loop stabilization.

## Figures and Tables

**Table 1:** Melting temperatures obtained in fits of the Boltzmann equation to the CD melting data of the studied DNA sequences.

Sequence	*T_m_*/°C
GCG5AG7	54 ± 3
GCG6AG7	58.9 ± 0.7
GCG6AG8	58.7 ± 0.4

**Table 2:** Fluorescence lifetimes (τ) and rotational correlation times (ρ) of ThT bound to GCG6AG7 and ThT bound to DNAh obtained by global analysis of the time-resolved fluorescence and anisotropy decays, respectively. The lifetime τ_1_ is a very short lifetime with low contribution to the observed fluorescence, which is attributed to a small proportion of ThT molecules undergoing TICT [52].

ThT	*τ*_1_/ns	*τ*_2_/ns	*τ*_3_*/*ns	*ρ* (no K^+^)/ns	*ρ* (with K^+^)/ns
GCG6AG7	0.40 ± 0.05	1.7 ± 0.2	4.5 ± 0.3	3.20 ± 0.07	5.6 ± 0.2
DNAh	0.39 ± 0.09	1.3 ± 0.2	-	3.6 ± 0.2	3.6 ± 0.2

## Data Availability

All relevant data are included in the manuscript and the Supplementary Data. Any other data are available from the authors on request.

## Supplementary Materials

The following are available online at https://www.mdpi.com/article/10.3390/ijms23020925/s1.
