# Supplementary material for: Spectroscopic Characterization of Mitochondrial G-Quadruplexes
Source: Int J Mol Sci. 2022 Jan 15;23(2):925. doi: 10.3390/ijms23020925 (PMC8780183; doi:10.3390/ijms23020925)
Supplement: Supplementary file 1 [file ijms-23-00925-s001.zip › ijms-1536116-supplementary.pdf]

Supplementary Information

Spectroscopic characterization of mitochondrial G-quadruplexes

Sara Illodo[1,2], Cibrán Pérez-González[1,2], Ramiro Barcia[3], Flor Rodríguez-Prieto[2], Wajih Al-Soufi[1], Mercedes Novo[1]*

[1] Departamento de Química Física, Facultade de Ciencias,
Universidade de Santiago de Compostela, Lugo, Spain

[2] Centro Singular de Investigación en Química Biolóxica e Materiais Moleculares (CiQUS),
Departamento de Química Física, Universidade de Santiago de Compostela,
Santiago de Compostela, Spain

[3] Departamento de Bioquímica e Bioloxía Molecular, Facultade de Veterinaria,
Universidade de Santiago de Compostela, Lugo, Spain

e-mail: m.novo@usc.es

Contents

Materials and Methods 2
Equations 3
Circular Dichroism data 4
Gel Electrophoresis data 7
References 7

Materials and Methods

Table S1. Abbreviations and descriptions of the nucleic acid sequences used in this work.

**Abbreviation**	**Type**	**Sequence (5' to 3')**
G5AG7	DNA	GGGGGAGGGGGGG
GCG5AG7	DNA	GCGGGGGAGGGGGGG
rGCG5AG5	RNA	GCGGGGGAGGGGGGG
G6AG7	DNA	GGGGGGAGGGGGGG
GCG6AG7	DNA	GCGGGGGGAGGGGGGG
rGCG6AG7	RNA	GCGGGGGGAGGGGGGG
G6AG8	DNA	GGGGGGAGGGGGGGG
GCG6AG8	DNA	GCGGGGGGAGGGGGGGG
rGCG6AG8	RNA	GCGGGGGGAGGGGGGGG
GCG6AG7 MUT	DNA	GCGGAGGGAGGAGGGG
GCG6AG8 MUT	DNA	GCGGAGGGAGGGAGGGG
hGCG6AG7	DNA	GCGGGGGGAGGGGGGGAAGACAGCACTCAGCAAGCA
hGCG6AG8	DNA	GCGGGGGGAGGGGGGGGAAGACAGCACTCAGCAAGCA
DNAh	DNA	TGCTTGCTGAGTGCTGTCAAGCGTCTCGTCCTGATGA
RNAh	RNA	UCAUCAGGACGAGACGCAAGGGAA

Two DNA:RNA G4 hybrids are formed between the non-template DNA (DNAh, green strand in Figure 1), the RNA transcript (RNAh, red strand in Figure 1) and the G4 sequence DNA (hGCG6AG7 or hGCG6AG8, blue strand in Figure 1):

- DNA:RNA-hGCG6AG7: DNAh hybridised with hGCG6AG7 and RNAh
- DNA:RNA-hGCG6AG8: DNAh hybridised with hGCG6AG8 and RNAh

Equations

Fluorescence Anisotropy

Both steady-state and time-resolved fluorescence anisotropy were calculated from the fluorescence intensity recorded with the emission polarizer oriented at 0° ($I_{\parallel}$) and 90° ($I_{\perp}$) with respect to the polarization direction of the excitation light, as shown in equation 1, where G is a correction factor for the dependency of the detection system on the polarization direction of the emitted light (1).

$$r = \frac{I_{\parallel} - G\, I_{\perp}}{I_{\parallel} + 2G\, I_{\perp}} \tag{S1}$$

Hill equation

Circular Dichroism titrations were analysed using the established semi-empiric *Hill's equation* (2). It describes the concentration dependence of the fraction of molecules bound to a given ligand. In our case, the sodium or potassium cations are the ligands and the nucleic acid experiments a change in topology due to the binding to the ligand, which can be described by the following equilibrium equation:

$$G4_i + cation \rightleftharpoons G4_f \tag{S2}$$

where $G4_i$ is the initially adopted topology and $G4_f$ the final topology.

Applying Hill's equation to this equilibrium, the following equation is obtained for the dependency of the molar ellipticity θ with cation concentration:

$$\theta = A\frac{K^n}{K^n + [cation]^n} + B\frac{[cation]^n}{K^n + [cation]^n} \tag{S3}$$

where K is the equilibrium constant of the backward process of topology reorganization described by equation (2) and n is the so called Hill's coefficient which provides a way to quantify the degree of cooperativity of the binding process. A and B account for the molar ellipticity of the two G4 topologies.

Boltzmann function

CD melting experiments' data were analysed using an empirical function, the modified *Boltzmann sigmoidal function*:

$$\theta = \theta_2 + \frac{\theta_1 - \theta_2}{1 + e^{\frac{T - T_m}{c}}} \tag{S4}$$

where θ_1 is the ellipticity value of the plateau at low temperatures and θ_2 the one at high temperatures, and c is a coefficient which indicates the steepness of the curve. The melting temperature (T_m) of the nucleic acid corresponds to the inflection point of the curve.

Circular Dichroism data

Figure S1 shows the CD spectra obtained for all studied DNA sequences in Phosphate buffer for different added salt concentrations.

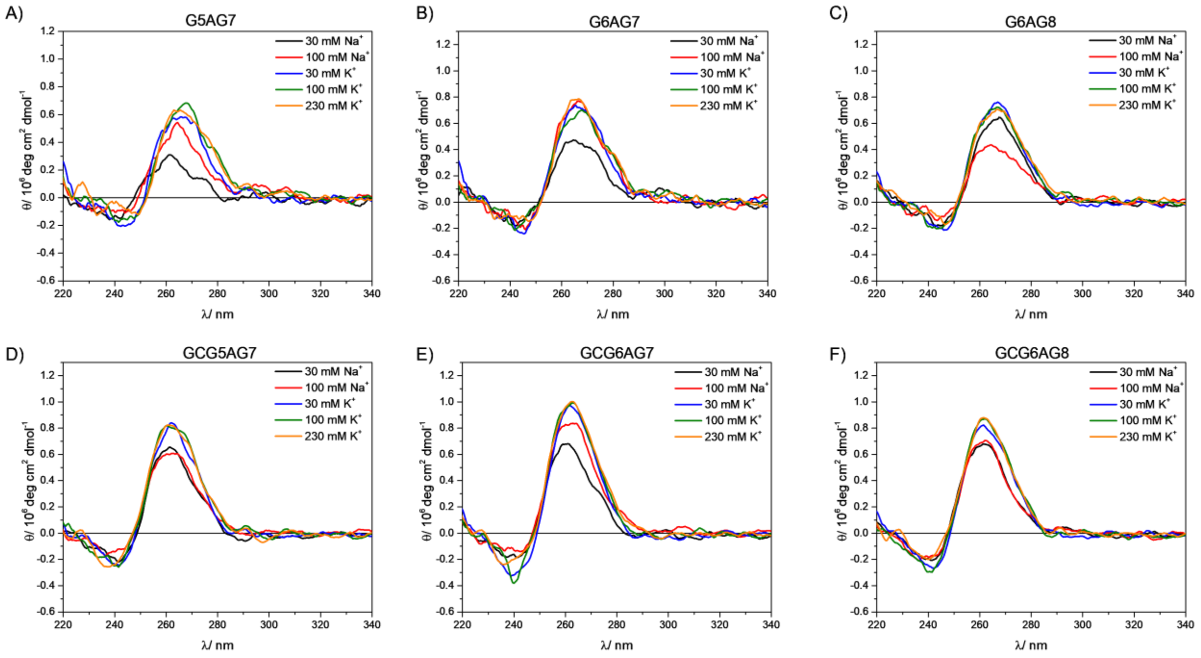

Figure S1. CD spectra for DNA sequences G5AG7, G6AG7, G6AG8, GCG5AG7, GCG6AG7 and GCG6AG8 in phosphate buffer with cations Na^+ and K^+ in the concentration range 30-230 mM.

Figure S2 shows the CD spectra obtained in the melting experiments at low and high potassium concentration.

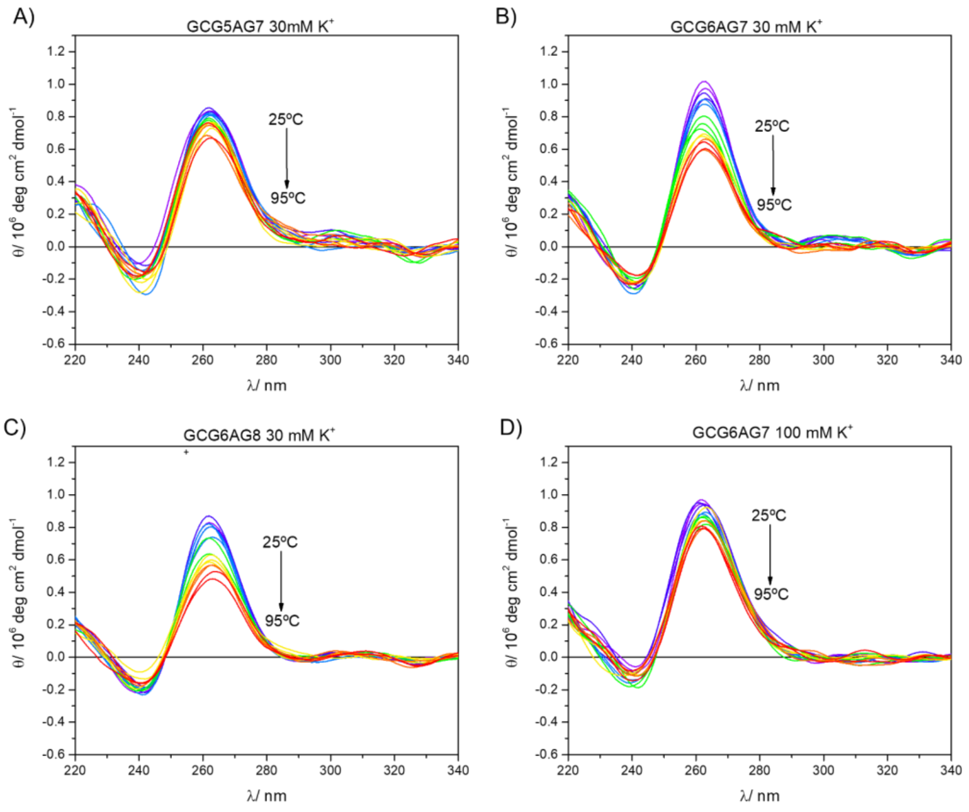

Figure S2. CD spectra obtained in the melting experiments in the temperature range from 25°C to 95°C.

Figure S3 shows the variation of the CD spectra of DNA sequences GCG6AG7 and GCG6AG8 with the concentrations of Na^+ and K^+ in Tris buffer, which allows the preparation of zero and low cation concentrations.

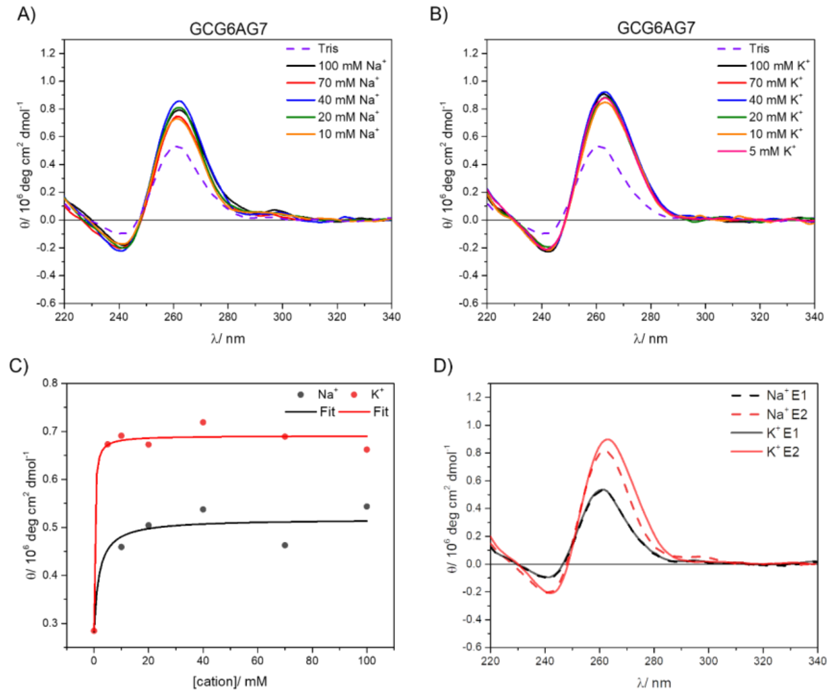

Figure S3. A) CD spectra of GCG6AG7 in Tris buffer at different added sodium concentrations. B) CD spectra of GCG6AG7 in Tris buffer at different added potassium concentrations. C) Experimental data and fitted curves using Hill's model of the intensities at the maxima for the CD spectra shown in A and B. D) Pure CD spectra of GCG6AG7 obtained from the non-linear global fit of Hill's model to the full CD spectra of potassium (continous line) and sodium (dashed line) titrations.

Figure S4 shows the CD spectra of the DNA sequences and the RNA sequences.

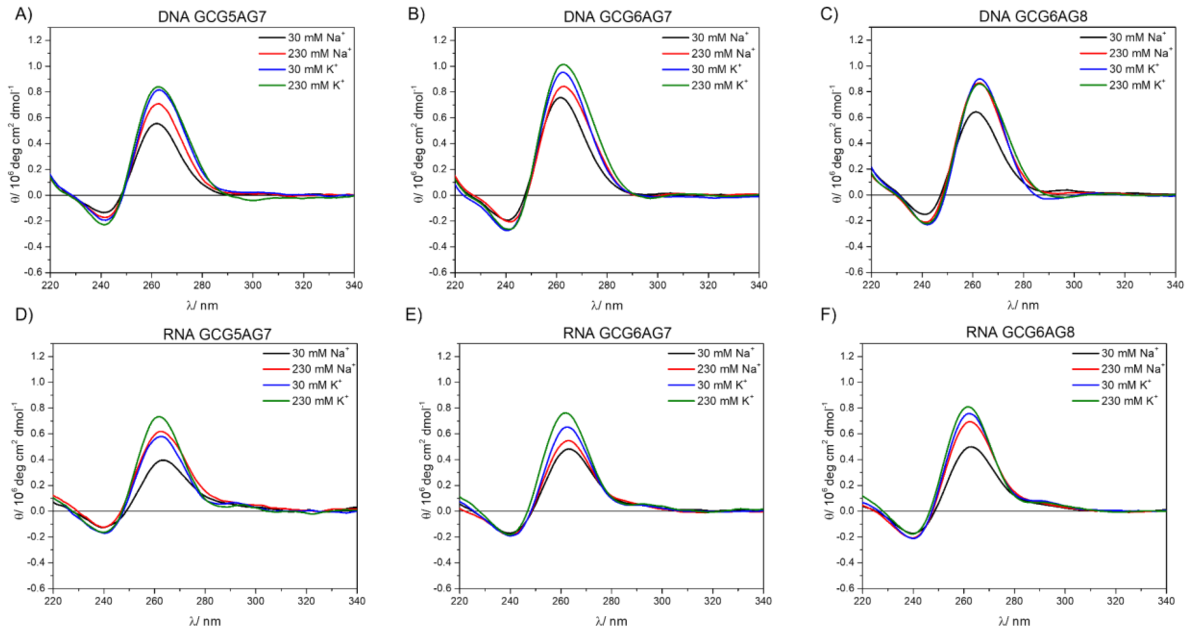

Figure S4. Comparison of the CD spectra of DNA and RNA sequences in the presence of a low and a high cation concentration.

CD spectra of DNA:RNA hybrid sequences were also acquired, but as shown in Figure SI5A and SI5B, the band at 260 nm was broader than usual. As DNA:RNA hybrid sequences are the result of hybridisation between three different sequences, and some unhybridized molecules could exist in the media, we subtracted from the DNA:RNA hybrid data the contribution of the non-G4 DNA band (DNAh). As shown in Figure SI5C, the subtraction of the ssDNA from the data results in a parallel G4 band.

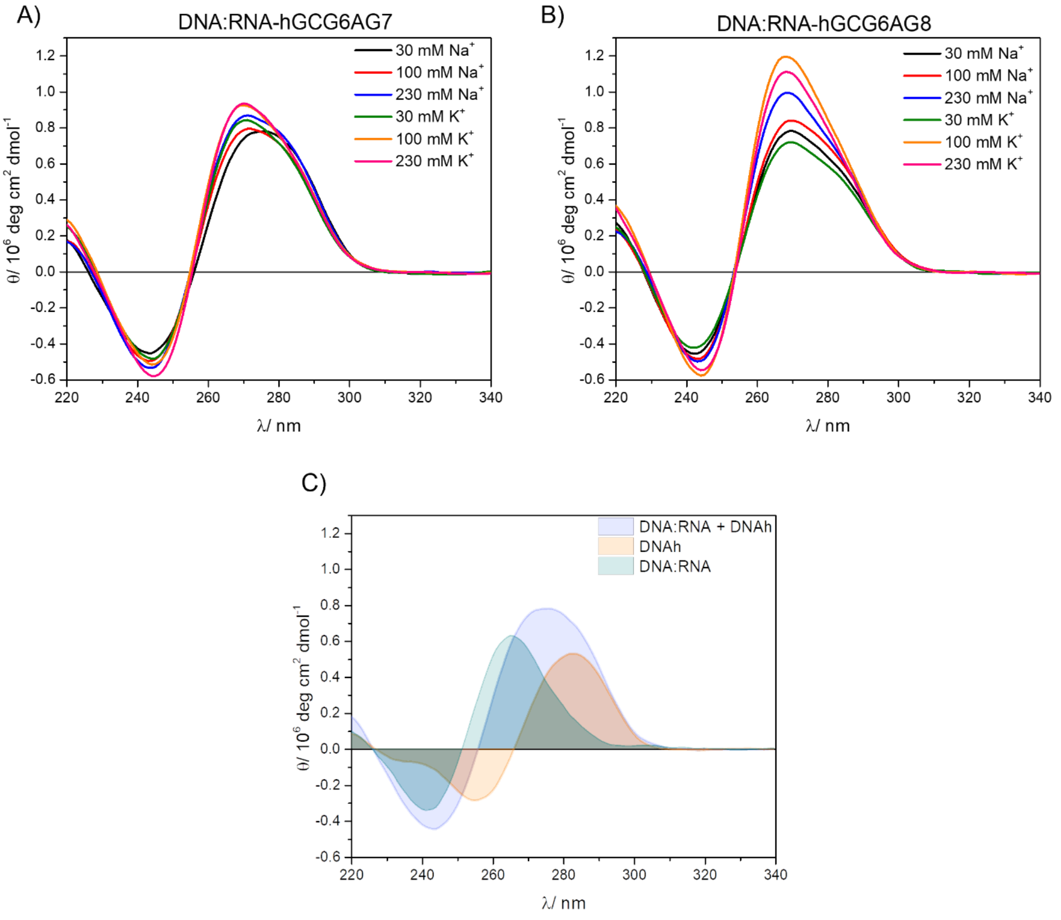

Figure S5. CD experimental spectra of DNA:RNA hybrids at different cation concentrations: A) hGCG6AG7; B) hGCG6AG8; C) Schematic representation of CD hybrid spectra analysis with ssDNA band substraction.

Gel Electrophoresis data

Figure S6 shows the same gel stained first with Sybr Gold and then, after washing out said stain, stained again with ThT. The same bands are observed in both gels, although ThT clearly shows much less intensity with non-G4 than with G4 structures. It can also be observed that the single chains hGCG6AG7 and hGCG6AG8 (see Table SI1), which can form G4s and have the same length as the non-G4 DNAh, show similar migrations.

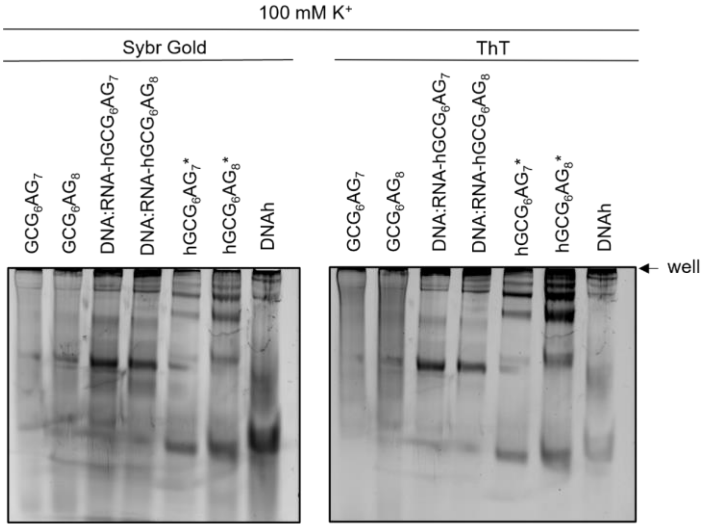

Figure S6. PAGE at 100 mM K^+ for the shown sequences stained first with Sybr Gold and then, after washing out said stain, stained again with ThT for comparison.

Table S2. Obtained retention factors (Rf), in percentage, for each sequence used in gel electrophoresis (Figure 8).

Sequence	Rf/ %	Sequence	Rf/ %
**GCG6AG7**	79	**DNA:RNA-hGCG6AG7 Tris**	37 / 64
**GCG6AG8**	77	**DNA:RNA-hGCG6AG8 Tris**	37 / 64
**DNAh**	58	**DNA:RNA-hGCG6AG7 K^+**	33
		**DNA:RNA-hGCG6AG8 K^+**	33

References

1. Lakowicz, J.R. (2006) Principles of Fluorescence Spectroscopy. Springer, USA.

2. Cantor, C.R. and Schimmel, P.R. (1980) Biophysical Chemistry: The Behavior of Biological Macromolecules. W.H. Freeman & Company, .
